# Acute lymphoblastic leukemia with clonal evolution due to delay in chemotherapy: A report of a case

**DOI:** 10.1002/jha2.483

**Published:** 2022-06-07

**Authors:** Ahmed Maseh Haidary, Ramin Saadaat, Jamshid Abdul‐Ghafar, Soma Rahmani, Sarah Noor, Sahar Noor, Najla Nasir, Maryam Ahmad, Ahmad Shekib Zahier, Rohullah Zahier, Haider Ali Malakzai, Abdul Sami Ibrahimkhil, Samuel Sharif, Tawab Baryali, Inamullah Mohib, Abdul Hadi Saqib, Raja Zahratul Azma

**Affiliations:** ^1^ Department of Pathology and Clinical Laboratory French Medical Institute for Mothers and Children Kabul Afghanistan; ^2^ Department of Haemato‐Oncology Ali Abad Hospital Kabul Afghanistan; ^3^ Department of Paediatric Medicine French Medical Institute for Mothers and Children Kabul Afghanistan; ^4^ Department of Internal Medicine Rabia Balkhi Hospital Kabul Afghanistan; ^5^ Department of Haemato‐Oncology Amiri Medical Complex Kabul Afghanistan; ^6^ Department of Internal Medicine Istiqlal Hospital Kabul Afghanistan; ^7^ Department of Quality Assurance French Medical Institute for Mothers and Children Kabul Afghanistan; ^8^ Faculty of Medicine Department of Pathology Universiti Kebangsaan Malaysia Kuala Lumpur Malaysia

**Keywords:** ALL, clonal evolution, delayed chemotherapy

## Abstract

Clonal evolution in acute leukemias is one of the most important factors that leads to therapeutic failure and disease relapse. Delay in therapeutic intervention is one of the reasons that leads toward clonal evolution. In this report, we present a case of acute lymphoblastic leukemia in which therapeutic delay resulted in clonal evolution that was detected by conventional karyotyping and was responsible for non‐responsiveness of the disease to conventional chemotherapy. A 17‐year‐old boy presented with generalized body aches, rapidly progressive pallor and lethargy. Bone marrow analysis was consistent with the diagnosis of B‐cell ALL. Karyotypic analysis revealed 46, XY male karyotype. The patient left the hospital due to financial reasons and after 40 days came back to the hospital. Repeated bone marrow analysis including cytogenetic studies revealed presence of three different clones of blast cells: one clone showed 46, XY with del(9p) and t (11;14), second clone showed 46, XY with del(7q) and del(9p), and the third clone showed 46, XY normal karyotype. The patient did not respond to chemotherapy and died within 1 week of induction chemotherapy (HyperCVAD‐A). Timely diagnosis and institution of chemotherapy in acute leukemias patients is the key to prevent clonal evolution and thus resistance of the disease to therapeutic interventions.

**Abbreviations**
ALL: Lymphoblastic leukemia
ALT: Alanine transaminase
APTT: Activated partial thromboplastin time
AST: Aspartate transaminase
BUN: Blood urea nitrogen
HCT: Hematocrit
HyperCVAD‐A: High‐dose cyclophosphamide‐based chemotherapeutic regime A containing cyclophosphamide, vincristine, adriamycin, and danorubicin
NGS: Next‐generation sequencing
PAX‐5: Paired box‐5 immunostain
PT: Prothrombin time

## 1 INTRODUCTION

Acute lymphoblastic leukemia (ALL) is the clonal proliferation of immature lymphoid precursors in the bone marrow and/or peripheral blood [1]. ALL has two main subclasses, which encompasses majority of the cases, that includes B‐cell ALL and T‐cell ALL [1]. The disease process is rapidly progressive and usually involves blood, bone marrow, and spleen, while sometimes non‐hematological sites can also be involved, especially with T‐cell ALL [1].

Over the past half century, there has been significant advances in the field of molecular biology that has led to better understanding of the pathophysiology of leukemias, including acute lymphoblastic leukemia [2]. Where in 1970s, the diagnosis of acute leukemias was based on clinical and morphological features of the blast cells, in today's era, the molecular‐cytogenetic modalities are used not only for diagnostic purposes but also for disease prognostication [3, 4]. Next‐generation sequencing and epigenetic studies are now being utilized to identify the molecular markers that can be targeted during therapy [1]. Many of the patients can now achieve complete remission with institution of agents aimed toward ablation or modification of specific molecular targets [1].

In spite of all the current modalities that are utilized for both diagnostic and prognostic purposes, a significant number of patients do not achieve the therapeutic targets [5]. Spontaneous clonal evolution results in emergence of clones of neoplastic cells that transforms a previously well‐controlled disease to one that is unresponsive to available therapeutic modalities [6]. It is very obvious that at cellular and molecular levels, there are many factors, sill unexplored, that take part in intercellular signaling and thus lead to clonal evolution [7].

Here we present an interesting case of ALL that underwent clonal evolution due to delay in institution of therapy, ultimately resulting in resistance of the disease to chemotherapeutic interventions.

## 2 CASE PRESENTATION

**FIGURE 1:**
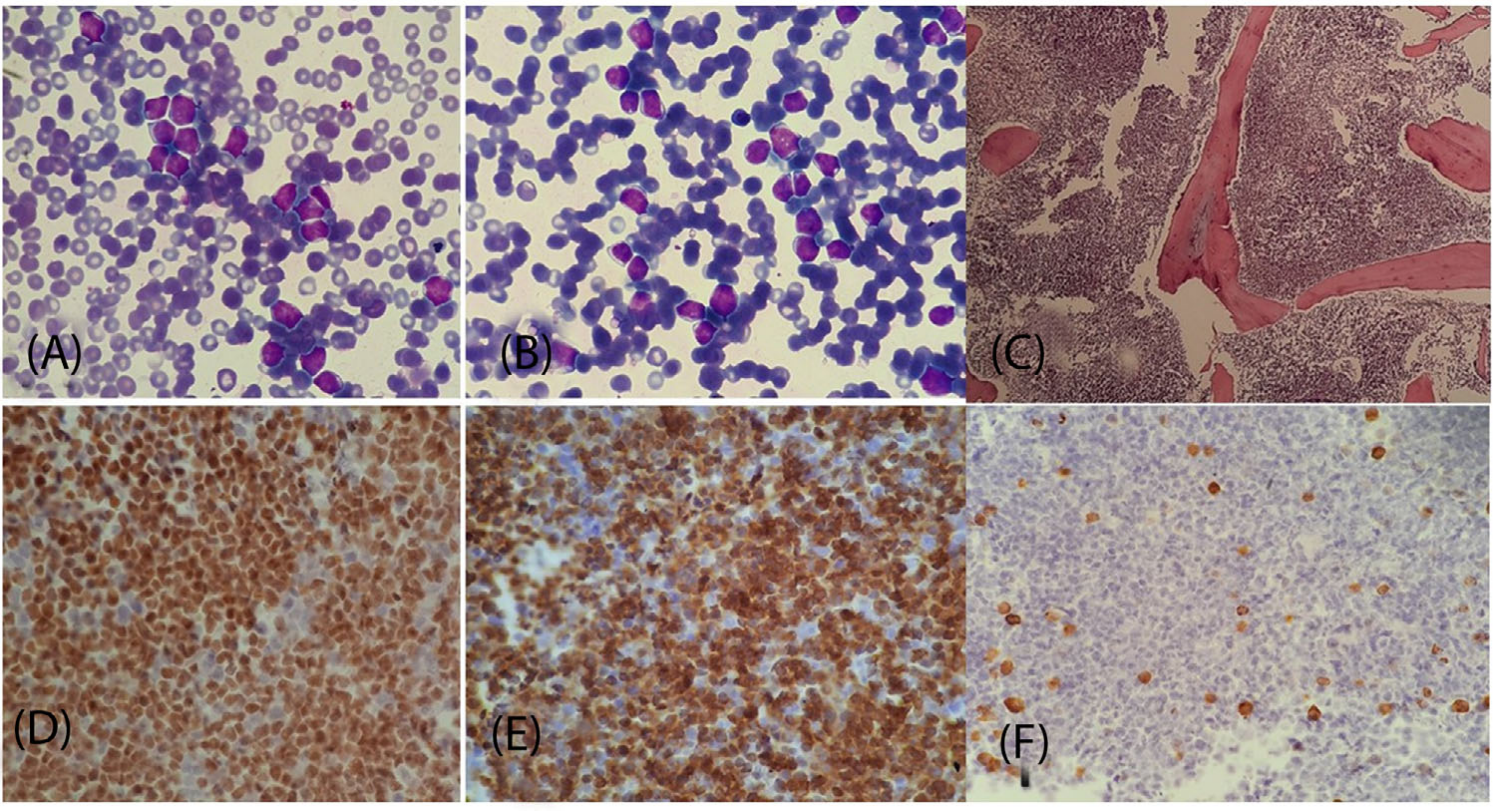
(A) Peripheral blood smear examination demonstrating presence of lymphoblasts. (B) Bone marrow aspirate smear preparation demonstrating presence of lymphoblasts. (C) Bone marrow trephine biopsy demonstrated diffuse infiltration by monomorphic lymphoblasts. Trephine immunohistochemistry demonstrated nuclear positivity of blast cells for PAX5 (D) and strong membrane positivity for CD20 (E), while CD3 identified the scattered residual T cells (F)

**FIGURE 2:**
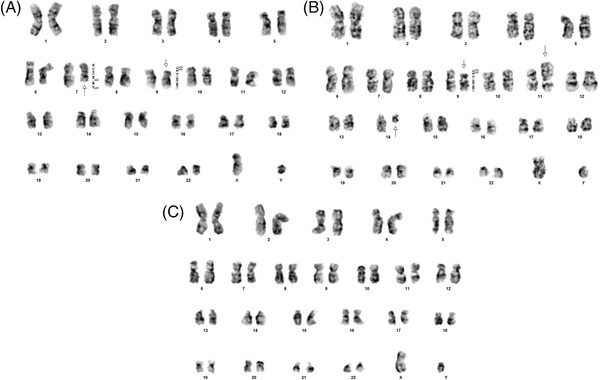
(A) [10 metaphases] 46, XY with deletion of chromosome 9 (p22; p24) t (11;14) (p15.3; q11.2); (B) [5 metaphases] showed 46, XY with deleletion of chromosome 7 (q22; q36) deletion of chromosome 9 (p22; p24); (C) [5 metaphases] showed 46, XY

A 17‐year‐old boy presented with generalized body aches, rapidly progressive pallor and lethargy, that had progressed over a duration of 4 weeks. Initial investigation demonstrated moderate anemia with moderate thrombocytopenia and presence of more than 65% blast cells in peripheral blood, while liver and renal profiles were unremarkable, as shown in Table 1. Bone marrow analysis was performed that demonstrated hypercellular marrow with presence of more than 90% blast cells that were strongly positive for PAX‐5 and CD20, shown in Figure 1, while they were negative for CD3. The blast cells were negative for Myeloperoxidase IHC, which is not shown in the figure. Cytogenetic analysis revealed 46, XY male karyotype without any structural chromosomal abnormalities. The patient left the hospital against medical advice due to financial reasons. Forty days after initial presentation, the patient turned back to the hospital. Parents of the patient informed that during this time the patient did not undergo any special therapeutic intervention and for supportive reasons 7 units of whole blood and 10 units of random donor platelets. Accordingly, the patient was found to be severely anemic and severely thrombocytopenic with peripheral blood film demonstrating more than 90% blast cells, also shown in Table 1. Accordingly, bone marrow analysis was repeated and demonstrated morphological and immunophenotypic features consistent with previous bone marrow analysis. Repeated cytogenetic analysis revealed presence of three different clones of blast cells: one clone showed 46, XY with del (p22; p24) t (11;14) (p15.3; q11.2) as shown in Figure 2A; second clone showed 46, XY with del [7] (q22; q36) del (p22; p24), as shown in Figure 2B; and the third clone of cells showed 46, XY, as shown in Figure 2C. The patient did not respond to chemotherapy and died within 1 week of induction chemotherapy (HyperCVAD‐A).

**TABLE 1:** Laboratory investigations performed during the initial as well as 40 days after diagnosis

Laboratory investigations				
	Parameters	At presentation	40th day post‐diagnosis	41st day postdiagnosis
Complete blood count	Hemoglobin	78 g/L	5.6 g/L	68 g/L
	Hematocrit	24.1%	17.5%	20.1%
	Total white cell count	21,000/μl	58,000/μl	30,600/μl
	Neutrophil	11%	5%	2%
	Blast cells	65%	>90%	>90%
	Platelet	71,000/μl	33,000/μl	26,000/μl
Coagulation profile	PT	11 s	15 s	13 s
	aPTT	28 s	33 s	30 s

Biochemistry				
Liver function test	AST	29 U/L	36 U/L	–
	ALT	33 U/L	45 U/L	–
	Total bilirubin	1.1 μmol/L	1.3	–
	Direct bilirubin	0.6 μmol/L	0.7 μmol/L	–
	Indirect bilirubin	0.5 μmol/L	0.6 μmol/L	–
Renal function Test	Creatinine	0.7 μmol/L	0.9 μmol/L	–
	BUN	18 μmol/L	21 μmol/L	–

HCT: hematocrit; PT: prothrombin time; APTT: activated partial thromboplastin time; AST: aspartate transaminase; ALT: alanine transaminase; BUN: blood urea nitrogen.

## 3 DISCUSSION

Pathogenesis of acute leukemia involves acquisition of genetic abnormalities either in the form of chromosomal aberrations or development of mutations, which in turn results in either functional loss or functional gain in the downstream signaling pathways at molecular level [3, 8]. The clone of leukemic cells that harbors such genetic abnormalities would grow uncontrollably, escaping the normal regulations of cell cycle [3, 8]. Novel therapeutic strategies are being developed with an effort to target the molecular determinants involved in the pathogenesis of neoplastic process [9].

New clone of cells can arise from the existing clone of leukemic cells, either spontaneously or during the institution of chemotherapy [8]. Evolved clone(s) of cells contain new genetic abnormalities either in the form of chromosomal aberrations in the form of abnormalities in chromosome number, chromosomal consistency, loss or gain of chromosomal regions, loss of whole chromosome or genetic mutations [6, 8]. Such genetic abnormalities in the new clone of neoplastic cells can transform an initially chemotherapy‐responsive acute leukemia to become chemotherapy‐resistant [10].

Recent evidence suggests that there are continuous intercellular communications between normal cells as well as cells in a neoplastic growth [11, 12]. It has been shown that such intercellular signaling plays important role in emergence of novel clone(s) in malignant disorders and therefore such signaling pathways could possibly be the therapeutic targets in the future [12].

Recent trails that utilized the next‐generation sequencing (NGS) studies for monitoring the clonal markers of acute leukemias identified that clonal evolution played significant role not only in clonal evolution but also in relapse of the disease and chemotherapeutic resistance [13]. It is possible that there are significant changes in the phenotype as well as cellular signaling machinery of neoplastic cells when they acquire novel genetic abnormalities, and thus they become unresponsive to chemotherapy. In our patient who initially presented with acute lymphoblastic leukemia with normal karyotype, the delay in institution of chemotherapy resulted in emergence of novel clones that rendered the disease resistant to chemotherapy.

It is clear that the early diagnosis, appropriate institution of diagnostic as well as prognostic modalities, early institution of chemotherapy, and if possible, implementation of targeted therapy are all essential steps to achieve complete remission. With this case, we want to emphasize upon the fact that in Afghanistan, there is a dire need not only for establishment of standard oncology institutions but also for health education of the public about various neoplastic processes and the importance of timely therapeutic intervention. Further multinational studies are required to elaborate about the role of intercellular pathways in clonal evolution and disease relapse. This would potentially allow for identification of novel therapeutic targets.

## 4 CONCLUSION

Our patient developed novel complex chromosomal abnormalities within the first month of diagnosis due to delay in chemotherapy. In our case report, we attempt to elaborate upon the fact that timely diagnosis and institution of therapy in cases of acute leukemia are indispensable to prevent emergence of therapy‐resistant leukemic clones.

## CONFLICT OF INTEREST

The authors declare they have no conflicts of interest.

## AUTHOR CONTRIBUTIONS

AMH, SRN, SHN, NJN, and RS conceived the idea. AMH, SRN, SHN, and ASZ were the major contributor to the writing of the manuscript. AHS, ASZ, AHS, and SS collected the laboratory data via integrated laboratory management system (ILMS). SS, AHS, INM, and MA performed the karyotypic analysis. AMH and SRN diagnosed the case. SHN provided the clinical information of the patient. AHS and SS performed cytogenetic studies. SRN, AMH, ASI, NJN, JAG and HAM were the major contributors for critically revising the manuscript for important intellectual content. JAG, NJN, SRN, and AMH have given expert opinion and final approval of the version to be published. All authors read and approved the final manuscript.

## CONSENT FOR PUBLICATION

Written informed consent was obtained from the patient's legal guardian for publication of this case report and any accompanying images. A copy of the written consent is available for review by the Editor‐in‐Chief of this journal.
